# Universal High-Resolution Copper Patterning on Diverse Substrates via Sequential Laser-Induced Transfer and Electroless Plating

**DOI:** 10.3390/mi17040391

**Published:** 2026-03-24

**Authors:** Yaqiang Ji, Juexuan Xu, Weibin Yin, Yuhao Huang, Ru Pan, Yiming Chen

**Affiliations:** 1 School of Mechanical Engineering, Dongguan University of Technology, Dongguan 523808, China; 17817207794@163.com (J.X.); yenwb537@163.com (W.Y.); 17825920153@163.com (Y.H.); panru_dgut@163.com (R.P.); 2 School of Chemistry and Materials Science, Hubei Engineering University, Xiaogan 432000, China

**Keywords:** laser-induced transfer, electroless copper plating, mechanical interlocking, flexible electronics

## Abstract

The fabrication of high-resolution and mechanically robust copper patterns remain a critical challenge in flexible electronics. Here, we present a universal metallization strategy that combines sequential two-step laser transfer, including laser-induced backward transfer and laser-induced forward transfer, with subsequent electroless copper plating. In this approach, laser-induced backward transfer first generates a transferable copper particle donor layer; subsequently, laser-induced forward transfer selectively embeds these catalytic copper particles into the surface of target substrates, constructing spatially confined activation networks while minimizing direct thermal exposure. These embedded seeds are then amplified into continuous copper conductors via electroless copper plating, achieving a high-resolution pattern (average minimum linewidth of approximately 20 μm) with robust interfacial integrity. Benefiting from laser-induced mechanical interlocking, the resulting copper patterns exhibit a low electrical resistivity of ~2.0 × 10^−8^ Ω·m (comparable to bulk copper) and maintain stable electromechanical performance even after 8000 bending cycles across a radius range of 3 to 6 mm. Furthermore, the fabricated versatile electrodes are successfully integrated into a triboelectric nanogenerator for tactile sensing and Morse code transmission. With its inherent substrate universality (e.g., polyimide, wood, fabric, and paper) and process scalability, this strategy provides a versatile route for manufacturing reliable copper electrodes in next-generation flexible electronic systems.

## 1. Introduction

Flexible electronics and wearable devices have attracted increasing attention in emerging areas such as human–machine interaction (HMI), soft robotics, health monitoring, and self-powered sensing [1,2,3,4]. The reliable operation of these systems critically depends on patterned electrodes that combine high electrical conductivity, fine resolution, and mechanical compliance [5,6,7]. Among various conductive materials, copper (Cu) is highly favored due to its high electrical conductivity, excellent resistance to electromigration, and significant cost advantages over precious metals such as gold and silver [8,9]. Although emerging gallium-based liquid metals [10,11] also offer notable cost benefits and possess high conductivity and high stretchability, concerns over leakage risks and biocompatibility also need to be taken into account. Therefore, achieving high-resolution and low-cost Cu patterning on diverse flexible polymers and unconventional substrates (including fabric, wood, and paper), while maintaining mechanical reliability under repeated deformation, remains a major challenge [12].

Conventional metallization strategies include photolithography, screen printing, inkjet printing, and electroless Cu plating (ECP) [13,14]. Photolithography offers submicron precision but relies on subtractive processes involving complex mask fabrication, high-cost equipment, and harsh chemical processing that are difficult to implement on rough or porous substrates [15,16,17]. Printing-based approaches follow additive routes but typically require metal inks containing organic binders, which reduce conductivity and necessitate high-temperature post-sintering, thereby limiting compatibility with heat-sensitive polymers [18,19]. Traditional ECP enables selective deposition in catalytic regions; however, it often depends on pre-patterned catalyst layers or noble-metal activation. This leads to additional processing complexity and potential issues of uneven catalyst distribution and insufficient interfacial adhesion [20]. Moreover, the intrinsic mechanical mismatch between rigid metal layers and soft substrates frequently results in cracking or delamination under cyclic bending, severely restricting long-term device stability [21].

To address the limitations of conventional manufacturing, laser direct writing (LDW) has emerged as a promising alternative due to its mask-free, non-contact, and programmable patterning capability [22,23,24]. It is worth noting that, unlike recent high-resolution methods that rely on pre-patterned stamps or masks to deposit copper nanoparticle seeds [25,26,27], laser-driven transfer techniques (such as LIFT/LIBT) offer greater flexibility in patterning and do not require consideration of pattern customization costs. Photochemical LDW can achieve high resolution through multiphoton processes, but its extremely low writing speed limits scalability [28,29]. Photothermal LDW improves throughput by locally heating precursors, yet uncontrolled thermal diffusion may degrade pattern fidelity and induce substrate damage [30,31]. Recently, laser-induced forward transfer (LIFT) and laser-induced backward transfer (LIBT) have been explored for localized material deposition across substrates [32,33]. Nevertheless, single-step laser transfer strategies remain constrained by the optical absorption and thermal stability of the target materials. High-energy irradiation may cause irreversible ablation or thermal penetration in heat-sensitive substrates such as PET and PI [34,35,36].

To overcome these critical limitations, we propose a universal patterning metallization strategy based on sequential two-step laser transfer (LIBT and LIFT) followed by ECP. In this approach, LIBT is first employed to pre-deposit a uniform Cu particle layer onto a glass donor, which serves as a transferable source. Subsequently, LIFT transfers selectively and embeds Cu particles into the surface of the target substrate, forming a spatially confined catalytic network. By separating catalyst generation from the final substrate, this donor–receiver configuration effectively minimizes direct thermal exposure of heat-sensitive materials. The subsequent ECP process then converts the embedded catalytic seeds into continuous Cu conductors with high resolution. In addition, the transient high-velocity impact during LIFT promotes partial particle embedding into the polymer matrix, enabling enhanced interfacial anchoring and mechanical robustness. Owing to its substrate versatility, this strategy not only enables customized metallization on diverse substrates (polyimide, wood, fabric, and paper) but also supports the fabrication of sliding-mode triboelectric nanogenerator (TENG) electrodes. Ultimately, it demonstrates significant potential for scalable and multifunctional flexible electronic systems.

## 2. Materials and Methods

### 2.1. Experimental Materials

PET films (thickness: 125 μm) were purchased from Dongguan Xinwang Plastic Materials Co., Ltd. (Dongguan, China) Ordinary glass slides (25 mm × 100 mm × 1 mm) were used as donor substrates. Pine wood substrates were cleaned with ethanol. Filter paper and fabric, as well as all other consumables, were purchased from Taobao (Hangzhou, China). The paper substrate used is qualitative cellulose filter paper (Grade 1 equivalent). Chemicals used for ECP, including copper sulfate pentahydrate (CuSO_4_·5H_2_O), potassium sodium tartrate tetrahydrate (KNaC_4_H_4_O_6_·4H_2_O), sodium hydroxide (NaOH), hydrochloric acid (HCl, 36–38% *w*/*w*), and ethylenediaminetetraacetic acid disodium (EDTA-2Na), were all purchased from Aladdin Industrial Corporation (Shanghai, China). All reagents were of analytical grade and used as received without further purification. Deionized water (resistivity: 18.2 MΩ·cm) was used throughout the experiments.

### 2.2. Sequential Two-Step Laser Transfer

The LIBT and LIFT processes were performed using a picosecond laser system (JPT, wavelength λ = 355 nm, maximum output power 10 W). During the LIBT process, the laser scanning position was controlled by a digital galvanometer according to a computer program, with laser processing parameters designed using EZCAD2 software. The processing was conducted in air on a clean glass slide, with a laser output power of 2 W, scanning speed of 1000 mm s^−1^, repetition rate of 40 kHz, and line spacing of 1 μm. The laser interacted with the local surface of the Cu layer, causing Cu particles to be sputtered in the reverse direction and redeposited on the glass surface, forming a continuous layer of Cu particles, which served as the consumable source for the subsequent LIFT process. In the LIFT process, Cu particles were selectively transferred from the glass donor to a receptor substrate (such as PET, PI, wood, fabric, or paper) by mechanically clamping the substrates face-to-face with a gap of approximately 100 μm. The laser, focused through the transparent glass onto the Cu particles, had an output power of 0.6 W, a scanning speed of 800 mm s^−1^, and a repetition rate of 400 kHz. The laser pulse energy propelled the Cu particles toward the receptor substrate, where high-momentum impacts caused the particles to be shallowly embedded in the substrate surface, forming a spatially confined distribution of catalytic precursor networks.

### 2.3. Metallization by ECP

After laser transfer, the substrate embedded with Cu particles was gently rinsed with deionized water to remove loose debris. Then, ECP was carried out in a water bath to maintain the temperature of the plating solution at 50 °C. The plating solution was freshly prepared before use by mixing Solution A and Solution B in a volume ratio of 10:1. Solution A: CuSO_4_·5H_2_O (14 g L^−1^), NaOH (14 g L^−1^), EDTA-2Na (20 g L^−1^), and KNaC_4_H_4_O_6_·4H_2_O (14 g L^−1^) dissolved in deionized water. Solution B: Formaldehyde solution (12 mL L^−1^). The typical deposition time for the PET substrate was 3–5 min to obtain a continuous Cu film. After deposition, the sample was thoroughly rinsed with deionized water to remove any residual plating solution from the surface.

### 2.4. Preparation and Testing of TENG

A sliding TENG was fabricated with Cu patterns as electrodes. This device consisted of a slider and a stator. The stator was based on PET, and the slider was a PVDF block. Cu comb-shaped electrodes (finger width: 3 mm, spacing: 1 mm, total 14 fingers) were prepared on PET through a two-step laser transfer combined with ECP. The electrical output performance of the TENG was tested using an electrometer. The slider was installed on a linear motor, and the sliding distance, speed, and acceleration were controlled. The open-circuit voltage under different mechanical motions was recorded.

### 2.5. Characterization

The morphology and microstructure of the samples were characterized by field emission scanning electron microscopy (FE-SEM, Zeiss, Oberkochen, Germany). The crystal structure was analyzed by X-ray diffraction (XRD, Bruker D8 Advance, Bruker, Ettlingen, Germany) using Cu Kα radiation (λ = 1.5406 Å). The wettability was evaluated using a contact angle meter (SDC-200GT, Sindin, Dongguan, China). The electrical resistance of the Cu patterns was measured using a source meter (Keithley 2450, Tektronix, Beaverton, OR, USA) in a four-probe configuration, and the electrical conductivity was calculated from the measured resistance and the sample dimensions. The adhesion of the Cu coatings was assessed by a cross-hatch tape test. The mechanical flexibility of the Cu patterns was evaluated by bending tests with real-time resistance monitoring.

## 3. Results and Discussion

**Figure 1:**
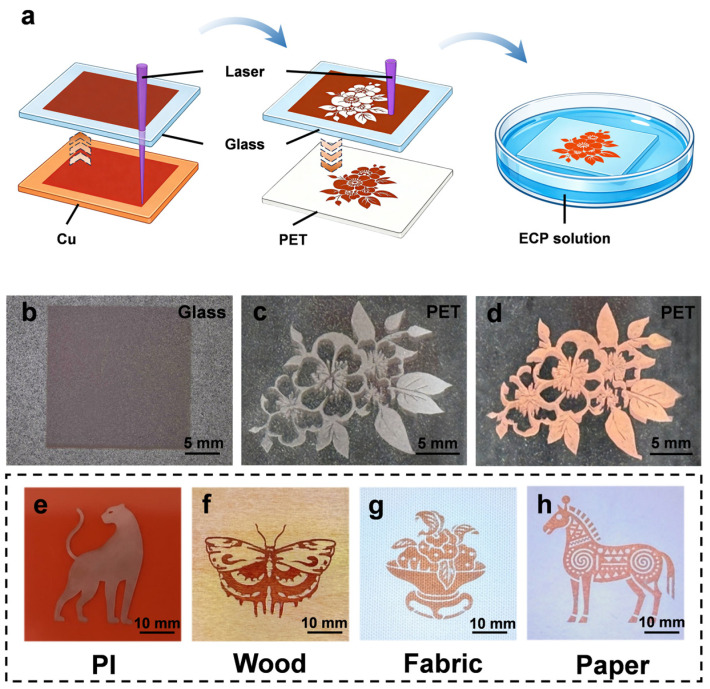
Schematic illustration of the two-step laser transfer fabrication strategy. (**a**) Overview of the fabrication process flow. (**b**) Optical image of the Cu donor layer formed on the glass substrate. (**c**) Cu seed layer transferred onto PET after the second laser step. (**d**) Final conductive Cu pattern on PET after ECP. (**e**–**h**) Optical photographs of Cu patterns fabricated on PI, wood, fabric, and paper.

Figure 1 shows the overall mechanism for constructing a patterned Cu conductive layer on a polymer substrate (such as PET) based on the combination of the sequential two-step laser transfer process (LIBT and LIFT) and subsequent ECP. Both transfer steps rely on localized absorption of laser energy by the Cu donor layer, which generates rapid heating and transient recoil/expansion forces that drive particle ejection. First, through LIBT, Cu particles generated by laser ablation are transferred and deposited onto the surface of a clean glass donor substrate, forming a spatially confined Cu donor layer. Under irradiation, the Cu layer locally absorbs the UV pulse energy, leading to rapid fragmentation or ablation and recoil-driven ejection of Cu species toward the incident side, where they are captured and redeposited on the adjacent glass surface. Through scanning, these ejected particles gradually accumulate into the quasi-continuous Cu donor layer for the subsequent transfer step. The macroscopic optical photograph (Figure 1b) shows that the pre-deposited layer exhibits uniform coloration and good surface continuity, indicating the good macroscopic uniformity, which provides a reliable foundation for the stability of the subsequent transfer process. Subsequently, LIFT is employed to selectively transfer the Cu particles from the glass substrate to the target receiver substrate (PET), as shown in Figure 1c. Because the transparent glass allows the laser to directly reach the donor/Cu interface, localized absorption generates a transient pressure impulse that launches Cu particles across the donor–receiver gap in the forward direction. Driven by the instantaneous momentum generated by the pulsed laser, the high-velocity Cu particles become embedded in the polymer surface layer and form stable mechanical anchoring, thereby constructing a catalytic seed layer for the subsequent electroless plating process. As deposition proceeds, the Cu nuclei gradually grow and coalesce, ultimately forming a continuous conductive Cu film (Figure 1d). It is worth noting that this strategy decouples the catalytic source generation from the receiver substrate selection within the processing pathway. This differs from the traditional method of directly laser-activating the substrate, which is often limited by the optical absorption characteristics and thermal stability of the substrate material. In contrast, the stepwise transfer strategy based on donor–acceptor separation is not limited by the substrate material, providing greater process flexibility for achieving universal metallization on various material surfaces [37,38]. To demonstrate this broad applicability, the optical photographs showcase high-quality Cu patterns successfully fabricated on diverse substrates, including polyimide (PI), wood, fabric, and paper, as shown in Figure 1e–h. During the electroless plating process, the Cu particles embedded in the surface of the receiver substrate act as catalytic active sites, promoting the autocatalytic reduction of Cu ions in the plating solution on their surfaces [39,40,41].

**Figure 2:**
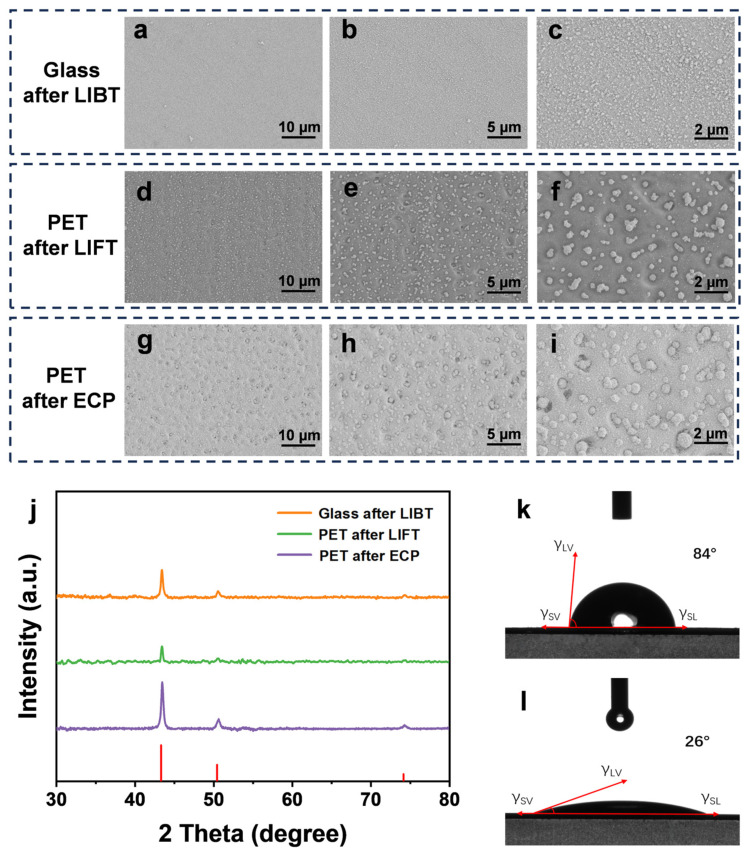
Microstructural evolution and compositional characterization during the fabrication process. (**a**–**c**) SEM images of the Cu donor layer on glass after LIBT at different magnifications. (**d**–**f**) SEM images of the Cu seed layer transferred onto PET after LIFT. (**g**–**i**) Surface morphology of the final Cu coating on PET after ECP. (**j**) XRD patterns of samples after LIBT (glass), LIFT (PET), and ECP (PET). The red vertical lines indicate the standard diffraction positions of FCC Cu. (**k**,**l**) Water contact angle measurements of pristine PET and laser-activated PET after LIFT.

In Figure 2, SEM was employed to systematically characterize the key interfacial structures, thereby clarifying the microstructural evolution at each stage of LIBT, LIFT, and ECP. As shown in Figure 2a–c, after LIBT treatment, a quasi-continuous Cu particle film was formed on the glass surface. This pre-deposited layer served as a donor layer for the subsequent LIFT process and was capable of secondary transfer. Unlike the donor films directly prepared in conventional LIFT, the donor layer in the present process was pre-generated via a preceding LIBT step. This intermediate layer functioned as a consumable transfer source, providing solid Cu particle material for the subsequent LIFT process. After further transfer onto the PET substrate via LIFT, the SEM images revealed that although partial melting led to an increase in particle size, the Cu particles remained relatively uniformly distributed and were tightly integrated with the polymer surface (Figure 2d–f). At higher magnification, the particle boundaries do not appear completely detached from the substrate background, but instead show morphology consistent with shallow embedding into the softened surface layer of PET under high-momentum impact. Such a surface-integrated configuration is indicative of localized anchoring sites, which can enhance the stability of the catalytic seed layer during the subsequent electroless plating process and strengthen interfacial bonding. This interpretation is consistent with the established role of surface topography and interlocking features in improving metal/polymer adhesion. The embedding mechanism assisted by the momentum generated from the ultraviolet pulsed laser enhanced the interfacial bonding between the catalytic sites and the target substrate, thereby improving the stability of the catalytic process during the subsequent electroless plating stage [42]. During electroless plating, the Cu particles previously transferred by LIFT acted as a seed layer to initiate the autocatalytic reduction of Cu ions in the plating solution. The initially discrete Cu nuclei gradually interconnected and eventually formed a continuous conductive Cu film (Figure 2g–i). Benefiting from the high purity and fully exposed surfaces of the transferred Cu particles, high catalytic activity was observed in the plating solution, enabling rapid initiation of autocatalytic Cu deposition and the formation of a continuous Cu layer [43]. This process demonstrates the rapid amplification effect of electroless plating on laser-transferred catalytic seeds, realizing the structural transformation from discrete micron-scale particles to a macroscopic continuous conductive layer within a short period. XRD characterization (Figure 2j) showed that after electroless plating, the sample exhibited characteristic diffraction peaks of face-centered cubic (FCC) metallic Cu (JCPDS No. 04-0836) at 2θ = 43°, 50°, and 74°, confirming the formation of a crystalline Cu phase [44]. In contrast, the diffraction signal of the pre-plated sample was significantly weakened and broadened, which can be attributed to the limited coherent scattering volume caused by the spatially discrete distribution of Cu particles after the second transfer. Meanwhile, contact angle measurements (Figure 2k,l) indicated that the wettability of the PET surface was significantly improved after laser treatment, suggesting an increase in apparent surface energy [44]. This enhancement facilitates uniform spreading of the electroless plating solution and promotes stronger interfacial bonding between the deposited metal layer and the substrate. In summary, LIBT generates transferable Cu catalytic particles, LIFT enables their transport and anchoring on arbitrary substrates, and electroless plating further transforms discrete catalytic seeds into a continuous conductive layer. Together, these processes establish a transfer-catalytic laser metallization mechanism, providing a structural foundation for subsequent high-resolution patterning and enhanced electromechanical performance.

**Figure 3:**
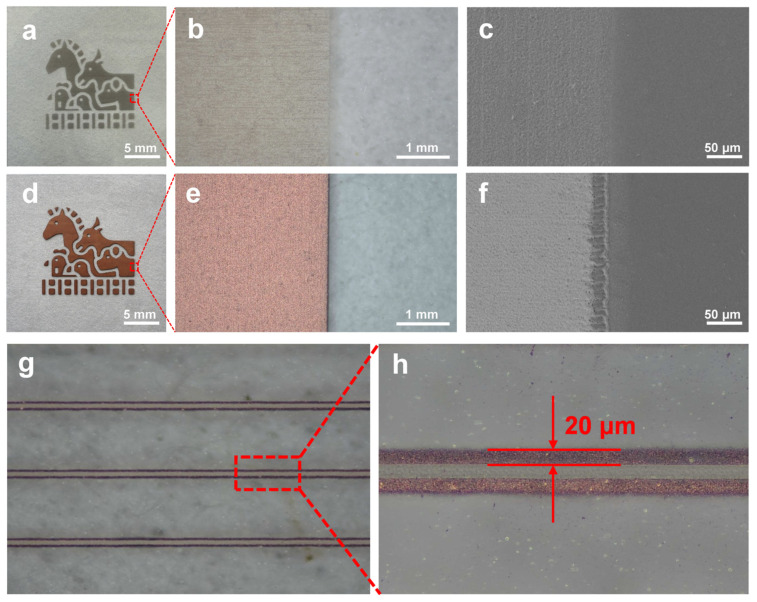
Morphological evolution and pattern resolution of Cu tracks on PET before and after ECP. (**a**–**c**) Digital photograph, optical micrograph, and SEM image of the Cu seed layer after LIFT. (**d**–**f**) Corresponding images after ECP. (**g**,**h**) Optical microscopy images showing the narrowest demonstrated Cu tracks on PET, for which an average minimum linewidth of approximately 20 μm.

We systematically investigated the evolution of the microscopic morphology and the patterning resolution of the prepared Cu electrodes to evaluate the device manufacturing quality and the film formation mechanism [45]. Figure 3a–f present a comparative analysis of the macroscopic appearance and microscopic morphology before and after ECP. In the initial stage, the laser-transferred seed layer on the PET surface appeared dark brown and was composed of discrete micron-sized particles (Figure 3a–c), indicating that Cu species were laser-ablated from the donor side and injected or embedded into the surface layer of the receiver substrate, thereby forming a porous catalytic framework. After electroless plating, the color of the circuit changed from dark brown to bright metallic orange (Figure 3d,e), suggesting the continuous reduction of Cu ions at the catalytic sites and progressive interconnection growth. High-magnification SEM further revealed the key transition from particle stacking to a dense continuous film. During ECP, deposition preferentially filled the gaps between the initial particles and established bridging (Figure 3c) [46], followed by lateral growth and coalescence that fused the discrete particles into a dense and cohesive metallic layer (Figure 3f). This film formation mechanism, characterized by “pore filling-coalescence densification,” plays a crucial role in establishing continuous electron transport pathways and minimizing interfacial scattering [47].

As shown in Figure 3g,h, the proposed two-step laser transfer strategy exhibited excellent pattern fidelity and processing precision. By optimizing the laser energy density and the donor–receiver spacing, continuous Cu circuits with linewidths down to approximately 20 μm were successfully fabricated on PET [48], featuring clear and sharp edges with highly consistent geometries. The pattern quality was found to be sensitive to several key laser-transfer parameters, particularly laser energy input, scanning speed, and donor–receiver gap. In the LIBT step, insufficient energy input (or excessively high scanning speed) led to an incomplete and non-uniform Cu donor layer, whereas excessive energy input caused over-ablation and deteriorated donor-layer continuity. In the subsequent LIFT step, overly low energy resulted in insufficient particle propulsion and sparse catalytic seed distribution, which was unfavorable for the formation of continuous Cu tracks during electroless plating. By contrast, excessive energy increased particle splashing, edge roughness, and the risk of local substrate damage. The donor–receiver gap also played an important role, an overly large gap reduced transfer precision because of particle dispersion during flight. Therefore, the selected LIBT/LIFT conditions represent a processing parameter that balances donor-layer integrity, particle embedding efficiency, and pattern fidelity, enabling the reproducible formation of continuous Cu circuits with clear edges and the average minimum linewidth of approximately 20 μm.

**Figure 4:**
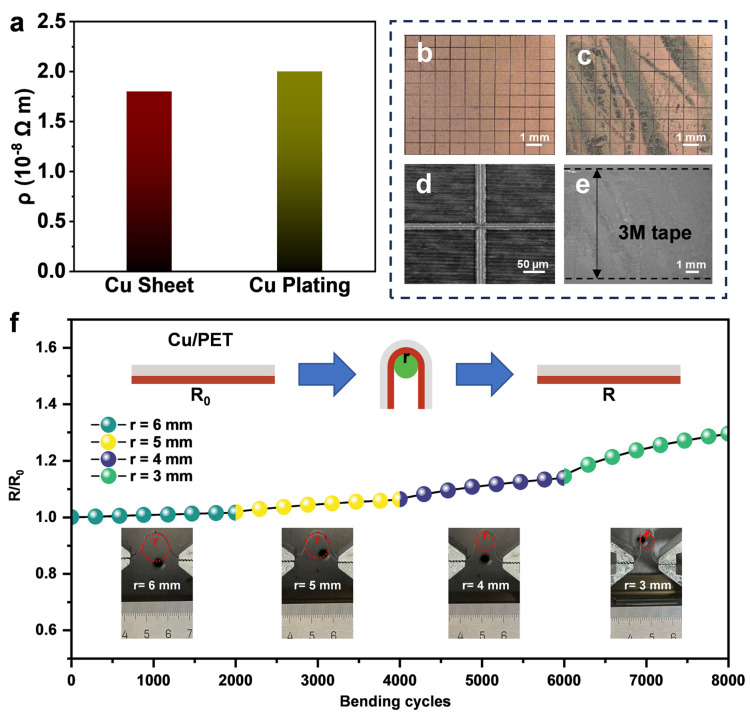
Electrical performance, adhesion behavior, and electromechanical durability of the ECP-Cu conductor on PET. (**a**) Electrical resistivity of the as-prepared Cu coating compared with commercial Cu sheet. (**b**,**c**) Optical images of the Cu surface before and after the standard tape adhesion test. (**d**) Cross-hatch pattern (1 mm × 1 mm) used for adhesion evaluation. (**e**) Surface of the peeled 3M tape after testing. (**f**) Normalized resistance change (R/R_0_) as a function of bending cycles at different bending radii.

We further conducted a quantitative evaluation of the electrical properties of the Cu layer, using a commercial Cu sheet as a reference. As shown in Figure 4a, the resistivity of the ECP-Cu layer was approximately 2.0 × 10^−8^ Ω·m, which is close to that of a commercial Cu sheet (approximately 1.8 × 10^−8^ Ω·m) [49]. This result indicates that the deposited Cu layer possesses high electrical conductivity, and that structural impediments to charge transport, such as grain boundaries, pores, and defects, are relatively limited [50]. The interfacial bonding stability was rigorously evaluated using a standard cross-cut tape test (ASTM D3359-23 [51]). As shown in Figure 4b,c, the Cu layer remained entirely intact after peeling, with an adhesion rating of the highest class 5B (0% detachment). High-magnification (Figure 4d) optical microscopy characterization further verifies that the patterned layer is completely severed, with no residual connections or unbroken links observed between the cut regions. Notably, no detectable transfer of the Cu layer was observed on the peeled 3M adhesive tape surface (Figure 4e); instead, residual adhesive from the tape remained on the Cu electrode surface. This observation suggests that the interfacial bonding strength between the Cu layer and the PET substrate exceeds the cohesive strength of the tape adhesive. The excellent adhesion stability can be attributed to the laser-induced embedding effect. In the second step of the process (LIFT), high-velocity particle impact drives Cu particles partially into the polymer matrix, forming a three-dimensional mechanical interlocking structure. Similar to a “root-anchoring” mechanism, this interlocking configuration significantly enhances the interfacial anti-peeling capability, thereby effectively suppressing electrode failure under external loading and repeated friction. Finally, to evaluate the electromechanical durability required for wearable applications, cyclic bending tests were performed. Figure 4f shows the normalized resistance variation (R/R_0_) of the Cu/PET conductor under different bending radii (3 to 6 mm). At larger bending radii (r ≥ 4 mm), the resistance remained essentially stable throughout the cycling process (R/R_0_ ≈ 1), indicating negligible bending fatigue effects. Even under the more severe condition of r = 3 mm, the resistance exhibited only a slight increase after 4000 cycles and remained below R/R_0_ < 1.3 after 8000 cycles, demonstrating good bending durability.

To further examine the structural stability after cyclic deformation, the Cu/PET conductor after 8000 bending cycles at the smallest bending radius (r = 3 mm) was additionally characterized by optical photographs and optical microscope photographs. As shown in Figure S1, the Cu track remained continuous after repeated bending, without fracture or peeling. The optical microscopy image further indicates that the patterned region remained intact, with clear boundaries and no cracking or delamination in the observed area. These post-bending observations are consistent with the relatively small resistance variation in Figure 4f, further confirming the good bending durability of the prepared Cu/PET conductor.

**Figure 5:**
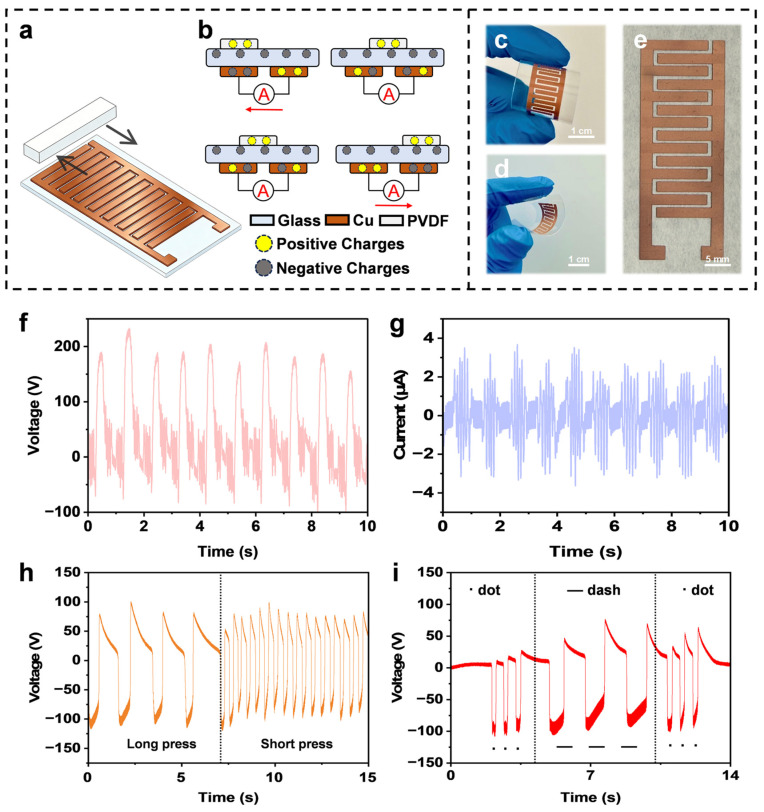
Application of the fabricated electrodes in a TENG-based self-powered sensor. (**a**) Schematic illustration of the TENG with polyethylene terephthalate (PET) substrate. (**b**) Schematic diagram illustrating the evolution of the electrostatic field distribution during the reciprocal motion of the slider. (**c**–**e**) Photographs of the as-prepared interdigital Cu electrodes. (**f**) Open-circuit voltage and (**g**) short-circuit current. (**h**) Output electrical signals generated by a self-powered sensor. (**i**) Demonstration of the triboelectric nanogenerator for Morse code transmission.

To further evaluate the practical application potential and operational stability of the prepared Cu electrodes, a sliding-mode TENG was constructed for self-powered sensing applications [52,53]. As shown in Figure 5a, the device employs a laser-transferred interdigital Cu electrode on a PET substrate as the fixed electrode, while an independent triboelectric layer serves as the sliding layer. Figure 5c,d present optical images of the device in a bent state. Even after significant and repeated bending deformation, no visible cracks, fractures, or delamination were observed in the electrode region. The magnified view in Figure 5e further shows that the boundaries of the interdigital structure remain clear and well preserved. Such mechanical robustness is likely associated with the firm interfacial anchoring between the transferred Cu particles and the polymer substrate. The working principle of the TENG is based on the coupling of contact electrification and electrostatic induction, as illustrated in Figure 5b. When the slider reciprocates over the interdigital electrode, periodic variations in the contact area generate a potential difference, thereby driving electrons to flow alternately between the electrodes through the external circuit. Figure 5f,g display reproducible open-circuit voltage and short-circuit current outputs during sliding motion, confirming stable electrical output under cyclic frictional excitation. In addition, as shown in Figure S2, after the repeated sliding test used for TENG output measurement, the interdigital Cu/PET electrode remained intact, without fracture or peeling. Optical inspection further indicated that the patterned region preserved clear boundaries, while only a slight resistance change was observed before and after testing, suggesting negligible degradation of the conductive pathway during repeated mechanical operation. These results support the good operational durability of the fabricated electrode and further strengthen its applicability in practical self-powered sensing.

The distinct signal responses under different mechanical stimuli also demonstrate the device’s capability to capture fine motion details. Specifically, as shown in Figure 5h, the TENG exhibits clearly distinguishable voltage waveforms corresponding to different actuation durations. Leveraging this sensitivity, the sensor was further demonstrated for Morse code transmission. As shown in Figure 5i, the device successfully transmitted the internationally recognized distress signal “SOS”. The recorded voltage waveforms clearly distinguish signal patterns according to the duration of the negative voltage peaks, where three narrow negative peaks correspond to short “dot” (S), whereas three wider peaks correspond to long “dash” (O). This result verifies the reliability of the prepared electrodes for tactile information encoding and emergency communication in HMI systems.

Although the TENG demonstrated here employs a macroscale interdigital geometry, it is used as a representative proof-of-concept device to verify the electrical functionality and mechanical durability of the fabricated Cu electrodes. Therefore, the aforementioned device demonstration should be understood as one representative example of this versatile metallization strategy rather than its sole application scenario. The broader significance of the present method lies in its ability to generate robust Cu patterns with an average minimum linewidth of approximately 20 μm on diverse substrates, including polymer, wood, fabric, and paper, while also providing a promising route for extending this process toward future micro/nanofabrication applications.

## 4. Conclusions

In summary, this work establishes a universal metallization strategy that integrates sequential LIBT and LIFT with ECP, enabling high-resolution copper patterning on diverse substrates. By decoupling catalyst generation from the receiver substrate, this donor–receptor configuration minimizes direct thermal exposure and extends compatibility to heat-sensitive and rough materials, including PET, PI, wood, fabric, and paper. The subsequent electroless amplification converts spatially confined catalytic seeds into continuous conductive networks with excellent pattern fidelity, achieving the average minimum linewidth of approximately 20 μm and a low electrical resistivity of ~2.0 × 10^−8^ Ω·m. Moreover, the high-velocity particle embedding during LIFT forms a mechanically interlocked interface. This “root-anchoring” structure substantially enhances adhesion and structural integrity, maintaining remarkable electromechanical stability even after 8000 bending cycles across a radius range of 3 to 6 mm. The successful integration of the resulting electrodes into a triboelectric nanogenerator for tactile sensing further validates the practical feasibility of this approach. Overall, this strategy offers a scalable and versatile route toward high-performance copper electrodes for multifunctional flexible electronic systems.

## Data Availability

The original contributions presented in this study are included in the article. Further inquiries can be directed to the corresponding authors.

## Supplementary Materials

The following supporting information can be downloaded at https://www.mdpi.com/article/10.3390/mi17040391/s1. Figure S1: Surface morphology of the sample after the bending test. Figure S2. Surface morphology of the sample after repeated sliding tests.
