# Supplementary material for: Universal High-Resolution Copper Patterning on Diverse Substrates via Sequential Laser-Induced Transfer and Electroless Plating
Source: Micromachines (Basel). 2026 Mar 24;17(4):391. doi: 10.3390/mi17040391 (PMC13117894; doi:10.3390/mi17040391)
Supplement: Supplementary file 1 [file micromachines-17-00391-s001.zip › micromachines-4200717-supplementary.pdf]

Supplementary Materials

Universal High-Resolution Copper Patterning on Diverse Substrates via Sequential Laser-Induced Transfer and Electroless Plating

Yaqiang Ji [1,*], Juexuan Xu [1], Weibin Yin [1], Yuhao Huang [1], Ru Pan [1] and Yiming Chen [2,*]

1 School of Mechanical Engineering, Dongguan University of Technology, Dongguan 523808, China; 17817207794@163.com (J.X.); yenwb537@163.com (W.Y.); 17825920153@163.com (Y.H.); panru_dgut@163.com (R.P.)
2 School of Chemistry and Materials Science, Hubei Engineering University, Xiaogan 432000, China
* Correspondence: jiyaqiang@dgut.edu.cn (Y.J.); yiming_chen@hbeu.edu.cn (Y.C.)

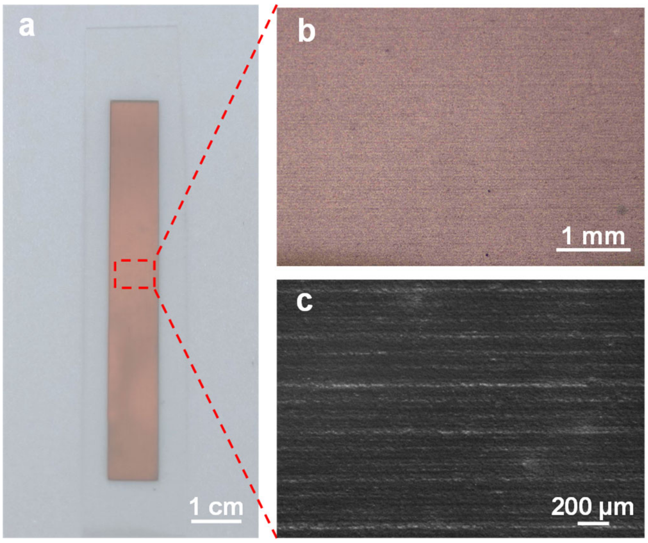

Figure S1. Surface morphology of the sample after the bending test. (**a**) Optical photograph of the sample after cyclic bending. Optical microscopy images of the corresponding bent region acquired under (**b**) reflected-light illumination and (**c**) transmitted-light illumination.

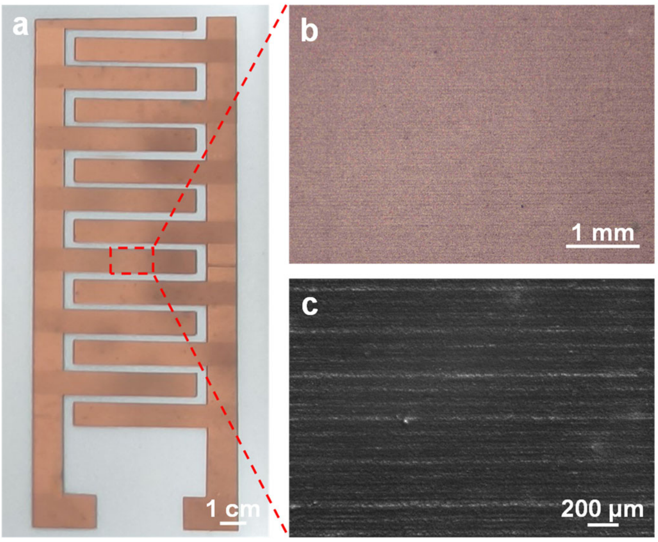

Figure S2. Surface morphology of the sample after repeated sliding tests. (**a**) Optical photograph of the sample after repeated sliding. Optical microscopy images of the corresponding sliding region acquired under (**b**) reflected-light illumination and (**c**) transmitted-light illumination.
